# Emerging Roles of p53 Family Members in Glucose Metabolism

**DOI:** 10.3390/ijms19030776

**Published:** 2018-03-08

**Authors:** Yoko Itahana, Koji Itahana

**Affiliations:** Cancer and Stem Cell Biology Program, Duke-NUS Medical School, 8 College Road, Singapore 169857, Singapore; yoko.itahana@duke-nus.edu.sg

**Keywords:** p53, p63, p73, p53 mutant, glucose metabolism, glycolysis, mitochondria, autophagy, diabetes, cancer

## Abstract

Glucose is the key source for most organisms to provide energy, as well as the key source for metabolites to generate building blocks in cells. The deregulation of glucose homeostasis occurs in various diseases, including the enhanced aerobic glycolysis that is observed in cancers, and insulin resistance in diabetes. Although p53 is thought to suppress tumorigenesis primarily by inducing cell cycle arrest, apoptosis, and senescence in response to stress, the non-canonical functions of p53 in cellular energy homeostasis and metabolism are also emerging as critical factors for tumor suppression. Increasing evidence suggests that p53 plays a significant role in regulating glucose homeostasis. Furthermore, the p53 family members p63 and p73, as well as gain-of-function p53 mutants, are also involved in glucose metabolism. Indeed, how this protein family regulates cellular energy levels is complicated and difficult to disentangle. This review discusses the roles of the p53 family in multiple metabolic processes, such as glycolysis, gluconeogenesis, aerobic respiration, and autophagy. We also discuss how the dysregulation of the p53 family in these processes leads to diseases such as cancer and diabetes. Elucidating the complexities of the p53 family members in glucose homeostasis will improve our understanding of these diseases.

## 1. Introduction

The well-known tumor suppressor p53 is mutated in about half of cancers. Tumor suppressor p53 is a transcription factor that responds to cellular damage and oncogenic insults, inducing cell cycle arrest, senescence, or apoptosis, depending on the cellular context. These canonical functions of p53 in somatic cells were thought to be sufficient to explain p53-mediated tumor suppression. However, recent studies challenge these long-held views of p53 function. For example, the p53 transactivation domain mutant p53^L25Q;W26S^ lacks the ability to induce cell cycle arrest and apoptosis triggered by DNA damage, but retains the ability to induce oncogene-induced senescence; knock-in mice expressing p53^L25Q;W26S^ display significant tumor suppression against oncogenic rat sarcoma viral oncogene homolog (RAS)-induced tumor formation [1]. Further, p53^K117R;K161R;K162R^ is mutated at three p53 acetylation sites and cannot induce cell cycle arrest, apoptosis, and senescence. However, unlike *p53*-null mice, p53^K117R;K161R;K162R^ knock-in mice do not show accelerated spontaneous tumor formation [2]. Interestingly, this p53^K117R;K161R;K162R^ retains the ability to induce non-canonical functions of p53, such as regulating metabolism and reducing reactive oxygen species (ROS) [2]. Lastly, the triple knockout mice that are deleted for *p21*, *p53-upregulated modulator of apoptosis* (*PUMA*) and *phorbol-12-myristate-13-acetate-induced protein 1* (*NOXA*/*PMAIP1*), which are important p53 target genes for inducing cell cycle arrest (*p21*) and apoptosis (*PUMA* and *NOXA*), are not more tumor-prone than wild-type mice [3]. These data suggest that the non-canonical functions of p53 can be critical for tumor suppression [4]. Recent evidence suggests that p53 regulates many genes in order to control metabolic processes, including glycolysis, oxidative phosphorylation, lipid metabolism, and ROS production, as well as amino acid, lipid, and nucleotide metabolism. Among these metabolic functions, this review focuses on the role of p53 in the metabolism of glucose, which is the central energy source of cells. We also discuss emerging insights into the functions of mutant p53, as well as the p53 family members p63 and p73, in glucose metabolism.

## 2. The Role of p53 in Glucose Metabolism

### 2.1. Glycolysis

**Figure 1:**
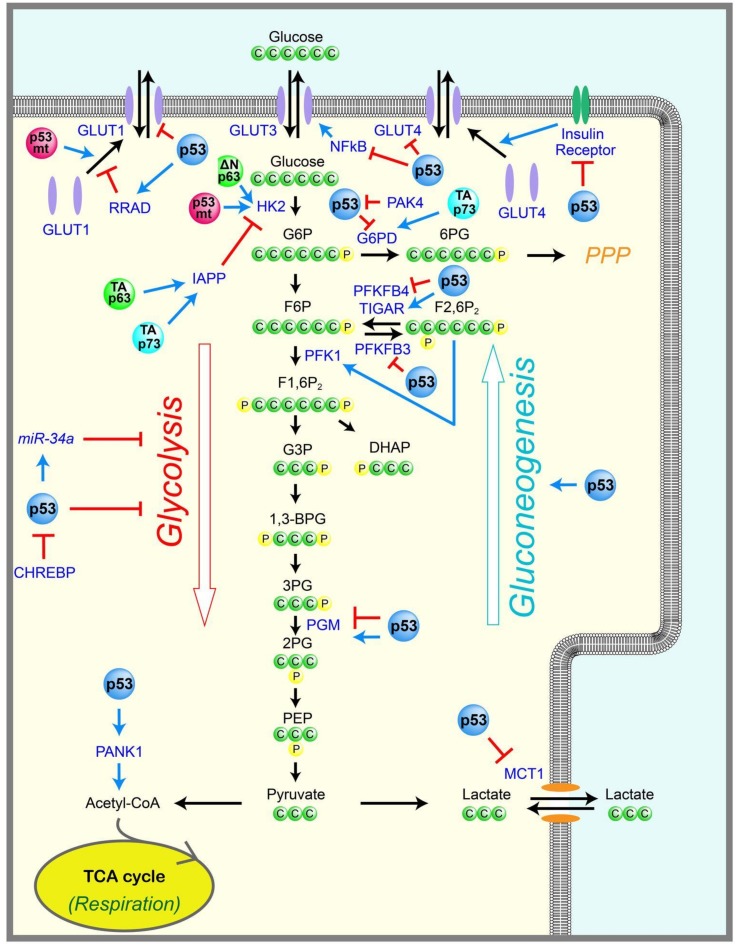
Regulation of glycolysis and gluconeogenesis by p53 family members and mutant p53. p53 inhibits glycolysis either by activating or repressing the transcriptions of genes or microRNAs involved in glycolysis, or by modulating the activity of the molecules involved in glycolysis in a transcription-independent manner. On the other hand, p53 promotes gluconeogenesis in a transcription-dependent manner. The mutant p53 and p53 family members p63 and p73 are also involved in the regulation of glycolysis. The positive regulations are shown in blue arrows, and the negative regulations are shown in red T-bar lines. The arrows in black indicate the conversion or the movement of molecules. C: carbon, P: phosphate, PPP: pentose phosphate pathway, G6P: glucose 6-phosphate, F6P: fructose 6-phosphate, F1,6P_2_: fructose 2,6-bisphosphate, G3P: glyceraldehyde 3-phosphate, 1,3-BPG: 1,3-bisphosphoglyceric acid, 3PG: 3-phosphoglycerate, 2PG: 2-phosphoglycerate, 6PG: 6-phosphogluconate, F2,6P_2_: fructose 2,6-bisphosphate, PEP: phosphoenolpyruvate, DHAP: dihydroxyacetone phosphate, TCA: tricarboxylic acid, GLUT: glucose transporter, IAPP: islet amyloid polypeptide, NFκB; nuclear factor kappa B, RRAD; *Ras-related glycolysis inhibitor and calcium channel regulator*, HK2: hexokinase 2, PAK4: p21-activated kinase 4, PFKFB: 6-phosphofructo-2-kinase/fructose-2,6-biphosphatase, G6PD: gucose-6-phosphate dehydrogenase, TIGAR: TP53-induced glycolysis and apoptosis regulator, PFK1: phosphofructokinase-1, PGM: phosphoglycerate mutase, MCT1: monocarboxylic acid transporter 1, PANK1: pantothenate kinases-1, CHREBP: carbohydrate responsive element binding protein, p53mt: p53 mutant, TAp63 and TAp73: full-length isoforms of p63 and p73, ΔNp63: the N-terminus deleted isoform of p63.

Glucose is the major cellular energy source, providing energy mostly in the form of adenosine triphosphate (ATP). Glucose uptake into cells is controlled mainly by the expression of the glucose transporters GLUT1-4. Once inside cells, glucose is converted into pyruvate via glycolysis in the cytoplasm, which generates two ATP molecules per glucose. If oxygen is available, pyruvate is oxidized in the mitochondria to generate 32–34 ATP per glucose through the tricarboxylic acid (TCA) cycle and oxidative phosphorylation. When oxygen is limited, pyruvate is converted into lactate via fermentation by lactate dehydrogenase in the cytoplasm (Figure 1). There is a complex interplay between oxidative phosphorylation and glycolysis, with reciprocal reinforcement depending on the amounts of oxygen, nutrients, cellular stresses, and genetic or epigenetic changes in cells. 

Interestingly, most cancer cells generate ATP using glycolysis, even when ample oxygen is available. This phenomenon, which is known as the “Warburg effect” [5,6,7], is a hallmark of cancer. Otto Warburg proposed that irreversible respiratory dysfunction in the mitochondria leads to aerobic glycolysis and cancer development. However, this hypothesis has been challenged recently by many studies showing that many cancer cells also use oxidative phosphorylation. It is still not well understood why aerobic glycolysis is enhanced in many types of cancer cells, given that oxidative phosphorylation produces greater amounts of ATP than glycolysis. One possible explanation is that lactate production from glucose may occur 10–100 times faster than the complete oxidation of glucose in the mitochondria [8]. It has been shown that ATP production in a given time is comparable between these two reactions [9]. Another possibility is that glycolysis supports the rapid growth of cancer cells by providing the precursors that are needed to synthesize building blocks, such as amino acids, nucleotides, lipids, other sugars, glycolipids, and glycoproteins. Whether the Warburg effect is a cause or consequence of cancer development is still controversial, and remains to be elucidated [5]. Non-canonical functions of p53, such as its roles in glycolysis inhibition, might attenuate or even prevent the Warburg effect. These could contribute to the tumor-suppressive functions of p53, given that enhanced glycolysis is a hallmark of cancer. 

p53 is involved in many steps of glycolysis, including glucose entry into cells (Figure 1). For example, p53 can suppress glucose transport by directly repressing the transcription of the glucose transporter genes, *GLUT1* and *GLUT4* [10], or by indirectly downregulating *GLUT3* expression via inhibition of nuclear factor kappa B (NF-κB) [11,12]. p53 can also suppress glucose transport by directly inducing transcription of *Ras-related glycolysis inhibitor and calcium channel regulator* (*RRAD*), which inhibits the translocation of GLUT1 at the plasma membrane [13]. Consistent with these results, RRAD overexpression inhibits glucose uptake in muscle and fat cells in culture [14]. Further, GLUT1 and GLUT3 are expressed highly in many types of cancers [15], which is consistent with a loss of p53-mediated repression. p53 can also inhibit glycolysis via the indirect inhibition of GLUT4. The insulin-induced activation of the insulin receptor triggers the translocation of GLUT4 from intracellular vesicles to the plasma membrane, and in turn, also triggers a rapid increase in glucose uptake. p53 represses the promoter of the *insulin receptor* (INSR) [16], thereby indirectly inhibiting glucose uptake by downregulating the insulin receptor. In addition to inhibiting glucose transport, p53 also inhibits the transport of lactate, the end product of fermentation, by repressing the lactate transporter, *monocarboxylic acid transporter 1* (*MCT1*), and leading to the accumulation of lactate that limits the glycolytic rate in cancer cells [17]. 

p53 also inhibits glycolysis by the induction of the “*TP53-induced glycolysis and apoptosis regulator*” (*TIGAR*), which dephosphorylates fructose-2,6-bisphosphate (F2,6P_2_) into fructose-6-phosphate (F6P) (Figure 1) [18]. Phosphofructokinase-1 (PFK1) catalyzes the conversion of F6P to fructose-1,6-bisphosphate (F1,6P_2_), an important rate-limiting step of glycolysis (Figure 1). F2,6P_2_ is a strong allosteric activator of PFK1. Therefore, TIGAR can reduce glycolysis by decreasing the levels of F2,6P_2_. p53 can also inhibit glycolysis by reducing the expression of the glycolytic enzyme phosphoglycerate mutase (PGM), which catalyzes the conversion of 3-phosphoglycerate (3PG) into 2-phosphoglycerate (2PG) during glycolysis, in a p53-mediated transcription-independent manner in fibroblasts (Figure 1) [19]. However, it is also reported that p53 directly transactivates the transcription of *PGM* in cardiac myocytes [20], suggesting that the p53-mediated regulation of PGM may be tissue dependent. In addition, p53 induces miR-34a, an inhibitor of several glycolytic enzymes [21]. Reciprocally, a glucose-responsive transcription factor—the carbohydrate responsive element binding protein (CHREBP)—was shown to enhance aerobic glycolysis partially via suppressing p53-mediated inhibition of glycolysis [22]. 

### 2.2. Gluconeogenesis

Contrary to glycolysis, which breaks down glucose and produces pyruvate, gluconeogenesis is the metabolic pathway that generates glucose from pyruvate (Figure 1). Emerging evidence suggests that p53 also regulates gluconeogenesis. Mice with an adipocyte-specific loss of p53 show a downregulation of genes that facilitate gluconeogenesis, such as the glucose-6-phosphatase catalytic subunit (G6PC), which converts glucose-6-phosphate (G6P) to glucose, and phosphoenolpyruvate carboxykinase-1 (PCK1), which converts oxaloacetate (OAA) to phosphoenolpyruvate (PEP) in the liver [23]. Consistent with this, a study of *p53*-null mice showed that p53 can induce gluconeogenesis and cell cycle arrest in the liver upon starvation, although prolonged starvation causes p53-mediated apoptosis, leading to severe liver atrophy [24]. Mechanistically, glucose removal induces peroxisome proliferator-activated receptor gamma coactivator-1 alpha (PGC-1α), which is a key inducer of mitochondrial biogenesis and gluconeogenesis in the liver, and directly binds to p53 and induces p53-dependent cell cycle arrest. However, prolonged glucose removal induces the degradation of PGC-1α, leading to p53 acetylation and p53-mediated apoptosis [24]. Another group evaluated a *p53*-null mouse model, and showed that p53 promotes gluconeogenesis by directly inducing *Pantothenate kinases-1* (*PANK1*), whose product catalyzes the first and rate-limiting step of coenzyme A (CoA) synthesis, which is critical for gluconeogenesis in the liver [25]. As expected, *PANK1*-knockout mice display impaired gluconeogenesis after starvation [26]. Similarly, it was shown that p53 stabilization, which is induced by starvation, is required for gluconeogenesis and amino acid catabolism in the liver [27]. p53 activation can also lead to glucose production in liver cells by inducing gluconeogenic enzymes, such as G6PC and phosphoenolpyruvate carboxykinase-2 (PCK2), as well as by supplying a gluconeogenic precursor, glycerol, via the induction of glycerol kinase (GK) or the glycerol transporters aquaporin 3 (AQP3) and aquaporin 9 (AQP9) [28]. 

### 2.3. Pentose Phosphate Pathway

p53 is also involved in the pentose phosphate pathway (PPP), a metabolic pathway that branches off from glycolysis (Figure 1). For example, *TIGAR* is induced by p53, leading to reduced F2,6P_2_ and decreased PFK1 activity, therefore preventing the conversion of F6P to F1,6P_2_ during glycolysis. The TIGAR-mediated accumulation of F6P promotes glucose flux toward PPP [18,29,30,31]. PPP generates metabolites used for the synthesis of nucleotides, nucleic acids, fatty acids, aromatic amino acids, and nicotinamide adenine dinucleotide phosphate hydrogen (NADPH). NADPH is necessary for regenerating glutathione (GSH), which is a major antioxidant that controls the cellular redox status. Therefore, TIGAR functions as an antioxidant to reduce the amounts of intracellular ROS [18,32]. Consistent with this, *TIGAR*-null mice are developmentally normal, but display defects in scavenging ROS and intestinal regeneration [33]. Further, p53 induces PPP and inhibits glycolysis by repressing the expression of *6-phosphofructo-2-kinase/fructose-2,6-biphosphatase 3* (*PFKFB3*) [34]. PFKFB3 is a bifunctional kinase/phosphatase that regulates the conversion between F6P and F2,6P_2_. Since the kinase activity of PFKFB3 is higher than its phosphatase activity, the p53-mediated inhibition of PFKFB3 results in decreased F2,6P_2_, which promotes PPP and inhibits glycolysis. In contrast to TIGAR and PFKFB3, cytoplasmic p53 has been shown to inhibit PPP and NADPH production by binding to and preventing the formation of the active dimer of glucose-6-phosphate dehydrogenase (G6PD), the rate-limiting enzyme of the PPP [35]. p21-activated kinase 4 (PAK4), a kinase that is frequently overexpressed in cancer, enhances the mouse double minute 2 (MDM2)-mediated degradation of p53. Therefore, PAK4 inhibits the ability of p53 to repress G6PD, resulting in the induction of PPP to support cancer growth [36]. It has also been shown that p53 inhibits PPP and enhances glycolysis by directly repressing the transcription of *PFKFB4* [37]. PFKFB4 is also a bifunctional kinase/phosphatase that is similar to PFKFB3, and regulates the conversion between F6P and F2,6P_2_. However, the phosphatase activity of PFKFB4 was shown to be slightly higher than the kinase activity, and the p53-mediated inhibition of PFKFB4 resulted in increased F2,6P_2_, leading to reduced PPP and enhanced glycolysis [37,38]. The ability of p53 to regulate the PPP may depend on cellular contexts and stresses, which remain to be elucidated.

### 2.4. Mitochondrial Metabolism

**Figure 2:**
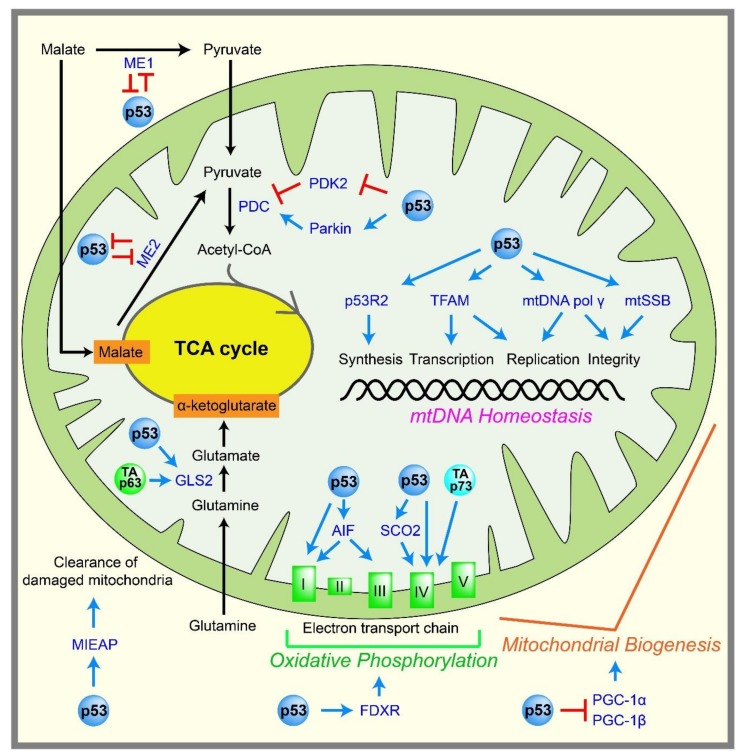
Regulation of mitochondrial metabolism by p53 family members. p53 enhances mitochondrial functions by inducing or repressing genes, or by interacting with proteins, both of which are involved in oxidative phosphorylation, the TCA cycle, and mitochondrial DNA (mtDNA) homeostasis. p63 and p73 are also involved in some of the steps in mitochondrial functions. The positive regulations are shown in blue arrows, and the negative regulations are shown in red T-bar lines. ME: malic enzyme, PDC: pyruvate dehydrogenase complex, PDK2: pyruvate dehydrogenase complex 2, TP53 inducible subunit M2B, TFAM: mitochondrial transcription factor A, mtSSB: mitochondrial single-strand binding protein, GLS2: glutaminase 2, AIF: apoptosis-inducing factor, SCO2: cytochrome *c* oxidase assembly protein, FDXR: ferredoxin reductase, PGC: peroxisome proliferator-activated receptor gamma coactivator, MIEAP: mitochondria-eating protein.

Pyruvate produced by glycolysis can enter the TCA cycle, which is coupled to oxidative phosphorylation to generate ATP in mitochondria. ATP is generated by the ATP synthase FoF1 complex, through using a proton gradient produced by the electron transport chain at the inner mitochondrial membrane (Figure 2). Mitochondria efficiently yield 32–34 ATP molecules during the aerobic oxidation of one glucose molecule. 

p53 is thought to promote oxidative phosphorylation and maintain mitochondrial integrity, either directly or indirectly (Figure 2). The activity of the mitochondrial complex IV is decreased in *p53*-knockout HCT116 cells compared with *p53* wild-type HCT116 cells [39]. In addition, mouse embryos lacking p53 display reduced amounts of ATP and complex IV [40]. Mechanistically, p53 directly activates the transcription of the *cytochrome c oxidase assembly protein* (*SCO2*), which is required for complex IV assembly [41], and the reduced oxidative phosphorylation in *p53*-null cells is rescued by reintroducing SCO2 expression to physiological levels [41,42].

Several other p53 target genes potentially promote oxidative phosphorylation. For instance, mutations in the p53 target gene *p53R2* (*RRM2B*), which encodes p53-controlled ribonucleotide reductase, cause the depletion of mitochondrial DNA (mtDNA) [43,44]. Furthermore, the loss of p53 results in decreased p53R2 expression, mitochondrial mass, and mtDNA copy numbers [45,46]. p53 can directly activate the transcription of the *mitochondrial transcription factor A* (*TFAM*) in order to increase the content of mtDNA [47]. p53 also directly induces the well-known apoptosis regulator, the *apoptosis-inducing factor* (*AIF*) [48]. *AIF*-deficient cells exhibit reduced complex I and III expression in the mitochondria, and reduced *AIF* expression leads to reduced oxidative phosphorylation in mice [49]. p53 also induces *ferredoxin reductase* (*FDXR*) [50], which is required for the biogenesis of iron–sulfur protein and heme, both of which are critical for oxidative phosphorylation. There is also a report that p53 indirectly induces the transcription of a gene encoding subunit I of complex IV [51]. p53 also induces *mitochondria-eating protein* (*MIEAP*), which promotes the elimination of oxidized proteins in mitochondria, as well as damaged mitochondria themselves, to support mitochondrial activity [52].

p53 also regulates the TCA cycle. p53 represses the promoter of *pyruvate dehydrogenase kinase 2* (*PDK2*) [53], which prevents acetyl-CoA production. PDK2 phosphorylates and inactivates the pyruvate dehydrogenase complex (PDC), which converts pyruvate to acetyl-CoA, the entry molecule for the TCA cycle. Therefore, p53 indirectly promotes the conversion of pyruvate into acetyl-CoA, leading to enhanced mitochondrial respiration. p53 can also stimulate transcription of the Parkinson’s disease-associated gene, *Parkin*. Parkin increases the protein expression of PDHA1, which is a component of PDC, leading to the increased mitochondrial respiration that indirectly limits glycolysis [54]. Malic enzymes ME1 and ME2, which convert malate to pyruvate, are known to adjust the TCA flux under different nutritional and growth conditions. Downregulation of ME1 and ME2 enzymes triggers p53 induction and cellular senescence; in turn, p53 can repress these genes to form a positive feedback loop. These findings suggest that p53 may function in part as a checkpoint protein to control the TCA cycle [55]. p53 also induces glutaminase 2 (GLS2), which is a key enzyme in the conversion of glutamine to glutamate, which is important for providing extra fuel to the TCA cycle and generating the antioxidant glutathione [56,57].

p53 promotes oxidative phosphorylation not only in the nucleus, functioning as a transcription factor, but also in the mitochondria. Besides the well-known function of p53 in enhancing apoptosis by translocating to the mitochondrial membrane [58], the constant presence of p53 in mitochondria helps to maintain mitochondrial integrity and support oxidative phosphorylation. For example, p53 maintains the stability of the mitochondrial genome inside the mitochondria by binding to the mtDNA polymerase gamma, enhancing its function in mtDNA replication [59], proofreading [60], and repair [61]. In addition, the interaction between mitochondrial single-strand binding protein (mtSSB) and p53 enhances the 3′→5′ exonuclease activity of p53 on mitochondrial DNA [62]. p53 inside the mitochondria also contributes to the incorporation [63] and the glycosylase steps [64] of the base excision repair of mtDNA. p53 binds to the mitochondrial transcription factor A (TFAM), which is critical for both mtDNA transcription and maintenance, and can enhance the binding between TFAM and cisplatin-damaged DNA, although the consequence of this enhanced binding is not yet clear [65]. Although p53 clearly functions in the mitochondria to promote mtDNA integrity, and in turn oxidative phosphorylation, how p53 passes through the outer and inner mitochondrial membranes to enter the mitochondrial matrix without an apparent mitochondrial targeting sequence remains to be elucidated. 

Although there is substantial evidence that suggests p53 enhances oxidative phosphorylation and maintains mitochondrial integrity, some reports challenge these concepts. For example, telomerase knockout mice show severe telomere dysfunction and p53 activation, leading to aging phenotypes, mitochondrial dysfunction, and reduced oxidative phosphorylation [66]. These changes are dependent on p53 activation, which triggers the repression of *peroxisome proliferator-activated receptor gamma coactivator 1 alpha* and *beta* (*PGC-1α and PGC-1β*), which are positive master regulators of mitochondrial biogenesis [66]. These data suggest that the p53-mediated regulation of oxidative phosphorylation and mitochondrial integrity may depend on cellular contexts. It is also noteworthy that reduced oxidative phosphorylation in *p53*-null cells is easily compensated by enhancing glycolysis to maintain the cellular ATP amounts [41]. The physiological impacts of the loss of p53 on oxidative phosphorylation and mitochondrial integrity may differ in tissues and surrounding cellular environments.

Overall, p53 seems to have contradictory roles in glucose metabolism. p53 inhibits glycolysis, which prevents the production of ATP and precursors for biosynthesis, but it also promotes oxidative phosphorylation and the TCA cycle, which produce ATP and precursors for biosynthesis, respectively. In addition, p53 enhances PPP flux and reduces ROS by inducing TIGAR. p53 is also known to reduce ROS by inducing GLUT9, which transports uric acid that is the one of the strongest antioxidants in cells [67]. However, p53 enhances oxidative phosphorylation, the major source of ROS. The effects of p53 on overall energy production, biosynthesis, and ROS may depend on the cell type, which influences the suite of p53 target genes. p53 may balance maintaining glucose homeostasis with ROS production. For example, under normal conditions, p53 maintains glucose homeostasis by inhibiting glycolysis while promoting oxidative phosphorylation to maximally generate 32–34 ATP by utilizing the low amount of pyruvate within mitochondria. Once glycolysis is inappropriately enhanced by oncogenic stress, the p53 that is activated by the stress might strongly inhibit glycolysis in order to prevent the Warburg effect. Although high ROS are toxic to cells, many signaling pathways also require ROS. Under unstressed conditions, the amounts of ROS may be produced through the p53-mediated induction of oxidative phosphorylation, while p53 acts as an antioxidant to prevent the accumulation of high levels of ROS. The complex roles of p53 in energy production and ROS remain to be elucidated. 

### 2.5. Autophagy

**Figure 3:**
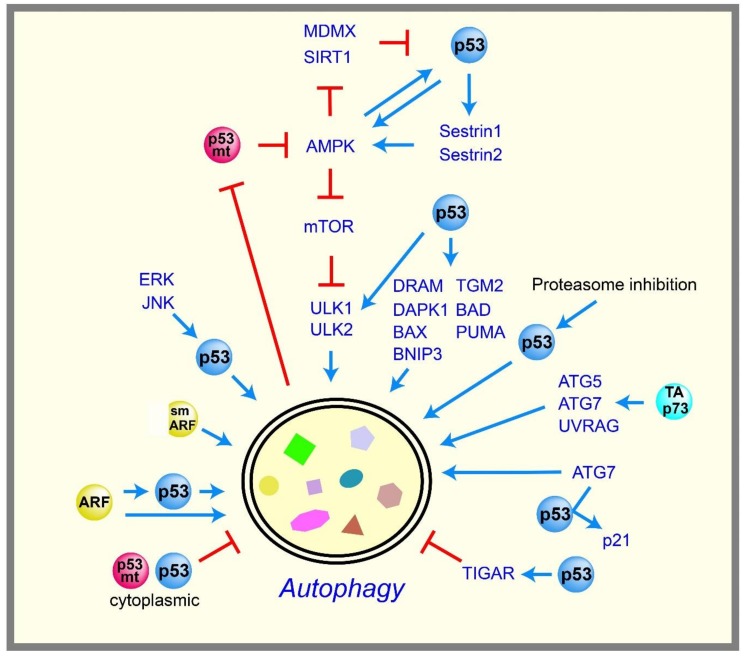
Regulation of autophagy by p53 family members and mutant p53. The regulation of autophagy by p53 is complex. p53 enhances autophagy by inducing distinct target genes, whereas cytoplasmic p53 inhibits autophagy. Alternative reading frame protein (ARF), an upstream regulator of p53, also enhances autophagy. A core autophagy regulator, ATG7, induces p21 via activating p53 through ATG7-p53 binding, contributing to cell survival during nutrient deprivation. p73 also induces multiple genes to enhance autophagy. On the other hand, mutant p53 prevents autophagy. The positive regulations are shown in blue arrows, and the negative regulations are shown in red T-bar lines. Double membrane structure describes an autophagosome containing cellular contents such as proteins, DNA, RNA, lipids, and small organelles that will be digested after fusion of autophagosome with lysosome. MDMX; mouse double minute X, SIRT1: sirtuin 1, AMPK: AMP-activated protein kinase, mTOR: mechanistic target of rapamycin, ULK: Unc-51-like kinase ERK: extracellular signal-related kinase, JNK: c-Jun N-terminal kinase, smARF: the short form of ARF, DRAM: damage-regulated autophagy modulator, DAPK1: death-associated protein kinase 1, BAD: BCL2 associated agonist of cell death, PUMA: p53 upregulated modulator of apoptosis, TGM2: transglutaminase 2, BNIP3: BCL2 interacting protein 3, BAX: BCL-2-associated X protein, ATG: autophagy protein, UVRAG: UV radiation resistance associated gene.

p53 controls glycolysis and mitochondrial metabolism to maintain glucose homeostasis in cells. If glucose is not available, cells can utilize other fuels, such as glutamine and lipid as energy sources, but the surrounding cellular environments may limit their availability. To compensate for this limitation, cells break down and utilize their own components as energy sources, such as proteins and organelles. This process is called “autophagy”, and consists of highly controlled catabolic steps that digest cytoplasmic components and organelles in lysosomes. Autophagy is also a tumor suppressive mechanism that removes unfolded proteins, damaged cellular components, and damaged organelles to maintain cellular homeostasis [68,69]. As a tumor suppressor, p53 is thought to positively regulate autophagy with a few exceptional cases (Figure 3). 

The AMP-activated protein kinase (AMPK) plays an essential role in cellular energy homeostasis, and is activated by decreased levels of intracellular ATP. AMPK inhibits mechanistic target of rapamycin (mTOR) kinase, which is a critical repressor of autophagy. It was reported that p53 enhances autophagy by inhibiting mTOR pathways via the activation of AMPK [70]. The genes encoding the β1 and β2 subunits of AMPK were subsequently confirmed as p53 targets [71]. p53 also induces Sestrin1 and Sestrin2, which activate AMPK [72,73]. Although these data suggest that AMPK acts downstream of p53, several reports suggest that AMPK also acts upstream of p53 to form a positive feedback loop, serving as a metabolic checkpoint to maintain energy homeostasis. For example, limited glucose conditions induce the phosphorylation of p53 via the activation of AMPK, thereby inducing cell cycle arrest to maintain energy homeostasis [74,75]. Activated AMPK also stabilizes p53 by phosphorylating and inactivating mouse double minute X (MDMX), which otherwise forms a complex with MDM2 to degrade p53 [76]. Similarly, AMPK also phosphorylates and inactivates the deacetylase sirtuin 1 (SIRT1), leading to p53 acetylation and stabilization [77]. 

p53 also promotes autophagy by inducing various autophagy-related genes, including *damage-regulated autophagy modulator* (*DRAM*), a lysosomal protein [78]; *death-associated protein kinase 1* (*DAPK1*), a kinase acting in the early steps of autophagy [79,80]; *unc-51-like kinase 1* and *2* (*ULK1* and *ULK2*), autophagy-initiation kinases downstream of mTOR [81]; and several pro-apoptotic Bcl-2 family members, such as *BCL2 associated agonist of cell death* (*BAD*) [82], *BCL-2-associated X protein* (*BAX*) [83], *PUMA* [83], and *BCL2 interacting protein 3* (*BNIP3*) [84,85]. In addition, p53 induces the late stage of autophagy by directly activating the transcription of *transglutaminase 2* (*TGM2*) to prevent oncogenic transformation [86]. p53 also participates in several signaling pathways that induce autophagy. For example, mitogen-activated protein kinase (MAPK) family proteins, such as extracellular signal-related kinase (ERK) and c-Jun N-terminal kinase (JNK), promote autophagy via p53 activation [87,88]. Proteasome inhibition also induces p53-dependent autophagy [89]. p53-mediated autophagy is also observed in vivo: restoring p53 with an inducible p53 fused with the hormone-binding domain of the modified estrogen receptor (ER) system in a Myc-induced lymphoma mouse model results in the induction of autophagy [90]. Full-length isoform of p73 (TAp73), a p53 family member, also induces autophagy by unknown mechanisms [91]. TAp73 is also known to induce the transcriptions of the autophagy-related genes *autophagy protein 5* (*ATG5*), *ATG7*, and *UV radiation resistance associated gene* (*UVRAG*) [92]. 

Consistent with the ability of p53 to induce autophagy, alternative reading frame protein (ARF), an upstream positive regulator of p53, also induces autophagy. Although full-length ARF is localized in both the nucleolus and mitochondria [93,94,95,96,97], the short form of ARF (smARF) is localized mainly in the mitochondria [98]. smARF induces autophagy in a p53-independent manner [98]; however, full-length ARF can induce autophagy via p53-dependent and independent mechanisms [94,99]. Nevertheless, the process by which ARF induces autophagy is not fully understood. 

Although many reports suggest that p53 promotes autophagy, several reports have challenged this concept. The inhibition of p53 can promote autophagy in human, mouse, and nematode cells, and the repression of autophagy is mediated by cytoplasmic, but not nuclear, p53 [100]. The p53-mediated repression of autophagy occurs in the G0/G1 phase [101]. The deficiency of the p53 ortholog gene *cep-1* in *C. elegans* promotes autophagy, leading to an increase of its lifespan [102]. In addition, the p53 target gene *TIGAR* inhibits autophagy by decreasing ROS upon nutrient deprivation [32], suggesting that nuclear p53 can also potentially inhibit autophagy. These data suggest that p53 can either promote or inhibit autophagy depending on the cellular contexts, and these contextual differences remain to be clarified. On the other hand, it has been proposed that p53 maintains better autophagic homeostasis by acting as a rheostat that adjusts the rate of autophagy depending on contexts of limited nutrient supply [103]. ATG7, an essential component of autophagy, can bind to p53 to induce cell cycle arrest through the induction of p21, contributing to cell survival during nutrient deprivation [104]. These findings suggest a reciprocal regulation between autophagy and p53, supporting the role of p53 in autophagic homeostasis. 

### 2.6. Diabetes

**Figure 4:**
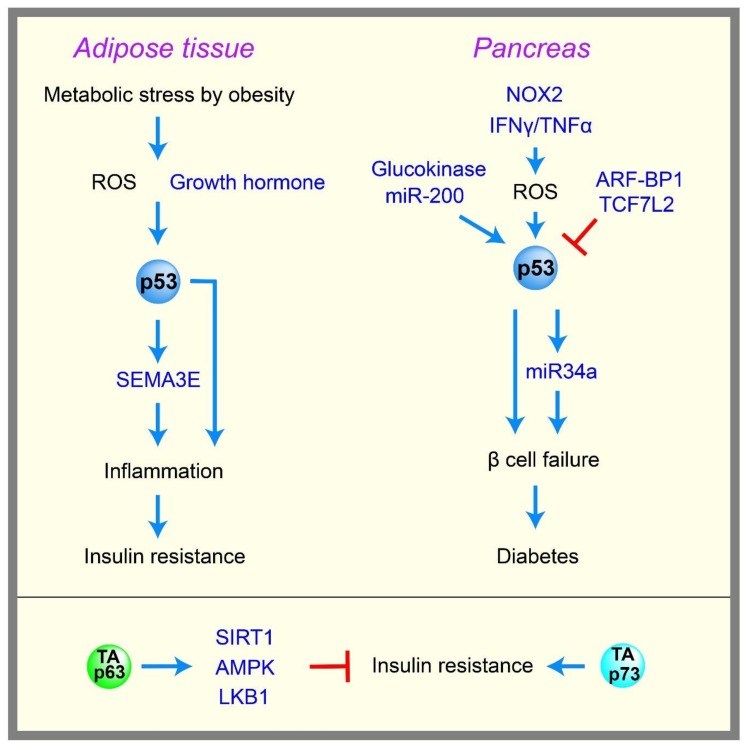
Involvements of p53 and p63 in diabetes. Activation of p53 can contribute to the development of insulin resistance and diabetes, whereas p63 prevents insulin resistance by inducing multiple target genes. The positive regulations are shown in blue arrows, and the negative regulations are shown in red T-bar lines. ROS: reactive oxygen species, NOX2; NADPH oxidase 2, IFNγ: interferon gamma, TNFα: tumor necrosis factor alpha, LKB1: liver kinase B1, SEMA3E: semaphorin 3E, ARF-BP1: ARF-Binding Protein 1, TCF7L2: T-cell factor 7-like 2.

Diabetes is defined as the dysregulation of glucose homeostasis, resulting in hyperglycemia. There are two types of diabetes, type 1 and type 2. Type 1 diabetes develops primarily in children and young adults, and is caused by an inability to produce enough insulin due to β-cell death in the pancreas, which is triggered mainly by autoimmune diseases. Type 2 diabetes generally develops in adults, and is caused by a failure to properly respond to insulin, called insulin resistance, and/or by impaired insulin secretion due to β-cell dysfunction/loss. Diabetes can increase the risk of cancer, and the common diabetes drug metformin is an anti-cancer drug [105]. As mentioned above, p53 controls glycolysis and mitochondrial metabolism by many different mechanisms; therefore, p53 may also be involved in diabetes, which many evidences suggest [106] (Figure 4). 

Obesity caused by a high-fat diet increases metabolic stress and ROS, which can activate p53 and thus trigger adipose tissue inflammation, senescence, and insulin resistance in type 2 diabetes. Specifically, p53 induces semaphorin 3E (Sema3E), which promotes macrophages infiltration and the inflammation of adipose tissue, leading to insulin resistance [107,108]. p53 is induced in the adipocytes of obese mice [109], and the inhibition of p53 activity in the adipose tissue of a mouse model of obesity and diabetes results in reduced senescence, decreased expression of proinflammatory cytokines, and recovery from insulin resistance in mice [23]. The growth hormone is reported to be required for increased p53 expression in adipocytes, and for insulin resistance in obese mice [110]. Liver dysfunction is also often associated with type 2 diabetes. p53 expression is induced in a mouse model of fatty liver disease [111], and a rat model of alcoholic liver disease [112]. The abrogation of p53 activity alleviates the diseases in a fatty liver mouse model [111,113]. 

Impaired insulin secretion due to the dysfunction/loss of pancreatic β cells significantly contributes to the development of both type 1 and type 2 diabetes. Many reports suggest that the induction of p53 in β cells triggered by metabolic stress enhances β cell failure and diabetes. For example, NADPH oxidase 2 (NOX2)-derived oxidative stress induces p53 and the apoptosis of β cell lines [114]. Co-treatment with tumor necrosis factor alpha (TNF-α) and interferon gamma (IFN-γ) induces p53, ROS, and apoptosis in β cell lines [115]. p53 induction in pancreatic β cells triggers miR34a expression, leading to sensitization to apoptosis [116]. Mice with an NHEJ (non-homologous end joining) deficiency undergo rapid lymphomagenesis, and a hypomorphic p53 mutant p53^R172P^ that lacks apoptotic function but retains the ability to induce senescence can rescue lymphomagenesis, but also induces severe diabetes accompanied with the senescence of pancreatic β cells [117]. Transgenic mice with an ectopic *p53* gene encoding Δ40p53 exhibit increased p53 activity in β cells, and develop diabetes with reduced pancreatic β cell mass [118]. Mice with a pancreatic β cell-specific deletion of *ARF-Binding Protein 1* (*ARF-BP1*), an E3 ligase for p53, display p53 stabilization and diabetic phenotypes [119]. The β cell-specific depletion of T-cell factor 7-like 2 (TCF7L2), a transcription factor that is known to support β cell survival, activates p53 and induces apoptosis in p53-dependent manner [120]. Cytosolic p53 induced by oxidative stress was shown to inhibit Parkin-mediated mitophagy and insulin secretion signals in the islet β cells of a mouse model of diabetes [121]. Hyperglycolysis in β cells triggered by chronic hyperglycemia or the genetic activation of glucokinase, as seen in patients with congenital hyperinsulinism, causes DNA double-strand breaks, followed by the activation of p53 and β cell failure [122]. miR-200 is induced in diabetic mice, and the β cell-specific overexpression of miR-200 induces diabetes due to the p53-mediated apoptosis of β cells [123]. Finally, the activation of p53 in β cells by the genetic deletion of its negative regulator, MDM2, inhibits glucose-stimulated insulin secretion, and induces glucose intolerance in mice via the impairment of mitochondrial respiration in β cells [124]. These data suggest that p53 activation by metabolic stress can promote diabetes in the adipose, liver, and pancreas. In addition, p53 may potentially contribute to global insulin resistance, which is a hallmark of type 2 diabetes. For example, p53 was shown to repress insulin receptor gene expression [16], and also limit glucose intake by repressing the expression of GLUT1 and GLUT3, as well as insulin-dependent GLUT4, as mentioned earlier. 

Although many reports suggest that p53 activity can contribute to diabetes, there are several studies showing that p53 can inhibit diabetes. For example, *p53*-knockout mice that are fed a high fat diet exhibit marked obesity and hepatic lipid accumulation compared with wild-type mice [125]. The activation of p53 by Nutlin-3 in a streptozotocin-induced diabetic mouse model reduces hyperglycemia [126]. Knock-in mice of p53 mutated at Ser18 (Ser15 for human), an important phosphorylation site for p53 activation, show insulin resistance [127]. Mice carrying an extra copy of the *p53* gene, “super p53” mice, exhibit improved glucose tolerance [128]. These contradictory data suggest that the role of p53 in diabetes could be dependent on the tissues and types of metabolic stresses that trigger p53 activity. Although more studies are needed to clarify this complexity, human population studies support a role for p53 in diabetes. A human polymorphism at codon 72 in p53 affects the ability of p53 to induce cell cycle arrest and apoptosis, and an arginine 72 polymorphism has been associated with type 1 and type 2 diabetes [129]. Consistent with these data, mice with the arginine 72 variant of p53 develop insulin resistance compared with mice with a proline 72 variant [130].

## 3. The Role of Mutant p53 in Glucose Metabolism

p53 is mutated in about half of the existing cancers. Many mutations are missense mutations in the DNA binding domain that not only abrogate DNA binding, but also act as dominant negatives that inhibit wild-type p53. Some mutant p53 proteins also show gain-of-functions to promote malignancy [131,132], such as inducing proliferation, metastasis, and chemoresistance by transactivating multiple genes [133,134] and by interacting with several binding partners [134,135]. Mutant p53 is overexpressed in many types of cancer cells, and may have a stoichiometrically greater degree of impact on particular metabolic processes than wild-type p53 [136]. 

Several reports showed that mutant p53 may have a role in glucose metabolism in cancers. When ample glucose is available, mutant p53 stimulates glycolysis by promoting GLUT1 translocation to the plasma membrane, which supports energy production and provides building blocks in cancer [137]. Mutant p53^G103S/E256G^, which retains DNA binding ability, is overexpressed in several liver cancer cell lines, and binds and activates the promoter of *hexokinase 2* (*HK2*) [138]. HK2 is upregulated in many types of cancers, and catalyzes the first step of glycolysis. On the other hand, when glucose is very limited, autophagy is induced; as a result, mutant p53 undergoes degradation by macroautophagy [139]. It was also shown that mutant p53 is degraded by chaperone-mediated autophagy when cells become confluent and stop proliferation [140]. However, mutant p53 can bind to the AMPK-α subunit, inhibiting AMPK activation [141], and the mutant p53 that is localized in the cytoplasm is also known to inhibit autophagy [142]. Therefore, the stability of mutant p53 may depend on the balance between autophagy signaling and the capacity of mutant p53 to inhibit autophagy. Tipping this balance toward autophagy by limiting glucose transport may have therapeutic potential by facilitating the degradation of mutant p53 to inhibit its gain-of-function properties.

## 4. The Role of p63 and p73 in Glucose Metabolism

p63 [143] and p73 [144] are homologues of p53, and share remarkable functional and structural similarities with p53 as transcription factors. However, unlike *p53*-knockout mice, *p63*- and *p73*-knockout mice exhibit severe developmental abnormalities, suggesting substantial functional diversity among the homologues [145]. Although p63 and p73 have multiple isoforms, there are two major isoforms of each protein (TAp63, ΔNp63, TAp73, and ΔNp73), which have independent promoters. Full-length isoforms TAp63 and TAp73 have a transactivation domain that is similar to that in p53. The N-terminus deleted isoforms ΔNp63 and ΔNp73 lack this domain and function, in part, as dominant-negative inhibitors of the full-length proteins. Recent results showed that both p63 and p73 participate in glucose metabolism, similar to p53; however, several reports suggest that some of their metabolic functions are the opposite of p53 [146,147,148]. For example, whereas p53 inhibits glucose uptake and induces fatty acid oxidation, *TAp63*-null mice exhibit insulin resistance, obesity, and glucose intolerance, all of which are associated with defects in glucose uptake in cells [149]. TAp63 is thought to prevent these symptoms by the transcriptional activation of *SIRT1*, *AMPKα2*, and *liver kinase B1* (*LKB1*), resulting in increased fatty acid synthesis and decreased fatty acid oxidation [149]. In contrast, *TAp73*-null mice show improved insulin sensitivity and glucose tolerance in mice fed a high-fat diet [150]. Although the role of p53 in PPP is still controversial, TAp73 increases the expression of G6PD, thereby enhancing PPP flux and supporting cancer cell proliferation [151,152]. 

On the other hand, there are some similarities between p53, TAp63, and TAp73. Similar to p53, TAp63 and TAp73 inhibit glycolysis, but by different mechanisms. TAp63 and TAp73 can induce islet amyloid polypeptide (IAPP), which is a peptide hormone from pancreatic β cells that can inhibit glycolysis through the inhibition of HK2 [153]. Furthermore, a synthetic analogue of IAPP, pramlintide can lead to tumor regression in *p53*-null mice [154]. Contrary to TAp63, ΔNp63 was shown to induce HK2 [153,155]. ΔNp63 was also shown to induce [156] or repress various enzymes that are involved in glucose metabolism by regulating the expression of miRNA [157,158]. Similar to p53, TAp63 can also induce GLS2, which provides extra fuel to the TCA cycle and reduces ROS [159]. *TAp73*-null mice show decreased oxygen consumption and mitochondrial complex IV activity [150], suggesting that TAp73 supports mitochondrial functions, similarly to p53. There are also mass spectrometry-based metabolomics studies showing overall metabolic functions of TAp63 and TAp73. TAp63 induces glycolysis and PPP [160], and TAp73 induces the TCA cycle and PPP [161]. Taken together, the current findings suggest that both TAp63 and TAp73 induce both PPP and the TCA cycle, whereas their effects on glycolysis depend on the cellular context. 

## 5. Conclusions and Future Directions

Glucose is the central energy source for most organisms, from single cell organisms such as bacteria and yeast, to multicellular organisms such as worms and humans. During the evolution from sea anemones to Homo sapiens, p53 family members acquired many cellular functions [162], including the regulation of glucose homeostasis, and their deregulation potentially leads to the development of diseases, such as cancer and diabetes. p53 family members induce or repress the transcription of many target genes that are involved in diverse pathways such as glycolysis, gluconeogenesis, mitochondrial metabolism—including the TCA cycle and oxidative phosphorylation, the AMPK energy sensor pathway, autophagy, and diabetes. Overall, p53 inhibits glycolysis, and enhances mitochondrial oxidative phosphorylation and the TCA cycle, whereas mutant p53 has the opposite effect. Contrary to the widely accepted beneficial role of p53, p53 can promote diabetes under conditions of metabolic stress, such as obesity, and inhibiting wild-type p53 can mitigate the development of diabetes. As discussed in this review, the role of p53 in glycolysis, mitochondrial regulation, and diabetes is controversial and contradictory. The complexities and inconsistencies in published articles regarding the effects of p53 could arise from the induction of different target genes in different cell types, in the absence or presence of distinct stresses. However, the process by which p53 chooses its target genes and decides the cell fate is quite complex, and still elusive [163,164]. Many factors have been proposed to contribute to the selection of target genes, such as p53 expression levels, posttranslational modifications, the presence of cofactors, and the sequence-dependent affinity of the p53 protein to the p53 binding element at the promoter of the p53 target genes. It was also shown that bivalent epigenetic modifications (dual markings with repressive histone H3 K27 trimethylation H3K27me3, and activating histone H3 K4 trimethylation H3K4me3) dictate the choice of p53 target genes in embryonic stem cells [165]. In addition, p53 can exert its effects by interacting with a myriad of binding partners in a transcription-independent manner [166]. Therefore, elucidating the complex p53 network in each cellular context and stimulus will help understand the development of diseases such as cancer and diabetes. Recent evidence suggests that p63, p73, and mutant p53 are also involved in glucose metabolism. p53, p63, and p73 have more than 10 isoforms, and can bind to each other, yielding greater complexity for regulating glucose homeostasis. For example, mutant p53 can bind and inhibit TAp63 and TAp73 [167]; however, the ability of mutant p53 to inhibit the metabolic functions of TAp63 and TAp73 is largely unknown. Several reports also showed that different missense mutations of p53 contribute to the different gain-of-function activities of mutant p53 [168,169]. Elucidating the complex metabolic network between p53, p63, p73, and mutant p53 is an important future direction. 

p53 induces TIGAR to promote PPP and generate GSH for reducing ROS, thus protecting cells from the genomic instability that is caused by oxidative stress. p53 induces autophagy to protect cells from damaged organelles and proteins. These p53 functions can prevent the initiation of tumor development by maintaining genomic stability. However, these functions can also promote cancer. The antioxidant function of p53 can protect cancers from apoptosis that is triggered by oxidative stress, drugs, and oncogenic activation. Autophagy also supports cancer survival in response to nutrient stress and chemotherapeutic drugs. Therefore, even the same functions of p53, such as autophagy and antioxidant functions, can lead to different outcomes for the human body. These complexities must be kept in mind in order to understand the effects of p53, depending on the stage of cancer and the tissues from which they are derived.
